# Verbal Descriptions Accompanying Numeric Information About the Risk: The Valence of Message and Linguistic Polarity

**DOI:** 10.1007/s10936-019-09666-7

**Published:** 2019-08-17

**Authors:** Agnieszka Olchowska-Kotala

**Affiliations:** Department of Medical Humanities and Social Science, Wroclaw Medical University, ul. Mikulicza-Radeckiego 7, 50-367 Wrocław, Poland

**Keywords:** Risk assessment, Verbal descriptions, Framing, Medical decisions, Indirect language, Valence

## Abstract

The aim of this study was to investigate the effect of presenting risk on decision making and evaluations with regard to the valence (positive vs. negative) and the linguistic polarity (direct vs. indirect). Participants were presented with a message in which patients were informed about risk associated with some diagnostic medical procedures. In the presented text, before obtaining statistical information about risk (e.g., 1 in 1000), four types of the verbal descriptions were used (risk is high/risk is not low/risk is low/risk is not high). The valence of information influenced the decision and respondent’s evaluation. Participants receiving a verbal description with a positive valence were more positive about the patient’ decision, and more positively evaluated the doctor and patient’s mood than participants receiving a description with a negative valence. Neither main nor interaction effects with linguistic polarity were found. The research does not support that indirect wording mitigates the meaning of a message on risk and highlights the strength and persistence of relying on the verbal description, particularly their affective valence.

## Introduction

Statistical data on the incidence of side effects of medical procedures may be useless if patients are not provided with professional assistance (Peters et al. 2009). If patients lack basic medical knowledge, they cannot properly interpret the information from their own doctors and, consequently, they expect it to be presented on a scale of good versus bad (Damman et al. 2016). Patients’ expectation that doctors should add verbal descriptions to numeric data has both advantages and disadvantages. On the one hand, this can significantly differentiate the subjective risk assessment (Knapp et al. 2015) and risk overestimation (Büchter et al. 2014; Berry et al. 2003), but, on the other hand, verbal information about risk seems to be a better predictor of individual preferences and behavioral intentions (Windschitl and Wells 1996).

Adding verbal descriptions of probability to numeric data may ‘frame’ patients’ understanding of risk (Knapp et al. 2015). Classical framing is defined as the presentation of two logically equivalent situations, where one is presented in positive or gain terms and the other in negative or loss terms (Tversky and Kahneman 1981). There are many studies on the effectiveness of gain- and loss-framed messages in promoting pro-health behaviors and making good medical decisions (Gallagher and Updegraff 2012; Gong et al. 2013). However, there is relatively little research regarding the differences between words and formulations in the same interpretational frame, such as “this is a bad message” versus “this message is not good”, or “this is a good message” versus “this message is not bad”. It is possible that messages about the incidence of side effects used in direct or indirect (negated) form e.g., “the risk is high” versus “the risk is not low” can significantly change the way in which patients perceive risk.

By analyzing the relation between indirect forms and a patient’s expectations, some researchers emphasize the fact that indirect forms suggest that an event is different, not typical or contrary to a patient’s expectations (Beukeboom et al. 2010; Stortenbeker 2016). Information presented indirectly impedes understanding (Burgers et al. 2015) and extends a patient’s response time (Christensen 2009).

According to the Gricean theory (Grice 1975) direct and negated-opposite terms do not have equivalent meanings and instead offer subtle differences in meaning. In psycholinguistics negation mitigates information and therefore the statement that something “is not pretty” is interpreted as “less than pretty”, not as “ugly” (Giora 2006), even if moderated by other factors, e.g., the confidence in the source (Betsch and Sachse 2013). Linguists note that the words “pretty” and “ugly” belong to the same, non-continuous and non-symmetrical scale, describing its opposite poles (Holleman and Maat 2009). In a series of experiments, Colston (1999) demonstrated that the symmetry of meaning does not always exist, i.e. the words “ugly” and “not ugly” do not always occupy the same distance from the middle of the scale.

Relevance theory, which attributes a significant role to the interlocutor’s expectations, attempts to explain this asymmetry of meaning (Wilson and Sperber 2004). According to this theory, when people expect a positive result, then defining this result as “bad” would mean the same as “not good” and defining the result as “good” would be more positive than “not bad”. This is opposite when we expect negative medical results or failed tests because then the information that the result “is not bad” means the same as “it is good”, whereas the information that the result “is not good” is less negative than “it is bad”.

Considering other theoretical frameworks, predictions about the effects of direct and indirect language look rather different. According to the rule of verbal politeness (Brown and Levinson 1987) irrespective of the receiver’s expectations or of the situation, the statement “not bad”, similarly to the Gricean theory, must indicate a different, slightly less positive evaluation than “good” (“good” > “not bad”), but indirect negative terms have a similar meaning to direct negative terms (“not good = bad”). This is because speakers often use indirect negative terms to express negative opinions (e.g., they say that something “is not good” instead of saying that “it is bad”). This is due to the fact that people are expected to speak in a socially positive manner and they “cooperate in maintaining face in interaction” (Brown and Levinson 1987, pp. 311).

Previous studies trying to verify which theory best describes relations between direct and indirect language did not lead to any unequivocal conclusions. Some studies confirmed symmetrical mitigation of information by using indirect forms (“good” > ”not bad” and “not good” > ”bad”) (Burgers et al. 2012), whereas other studies, according to the rule of verbal politeness, indicate no symmetry of meaning for direct and indirect positive terms (“good” > ”not bad”, but “not bad” = ”bad”) (Kamoen et al. 2015). Other results can be best explained by including theory of verbal politeness into the theory of relevance (positive expectation: “good” > ”not good” and “not good” = ”bad”; negative expectation: “good” = ”not bad” and “not good” = ”bad”) (Colston 1999).

Studies on the symmetry of meaning are related to different aspects of life. Due to the fact that this study pertains to making health-related decisions and presenting negative information, we formulated hypotheses according to the previous results in the area of health, which assumed that information is mitigated by the means of indirect forms (Burgers et al. 2012). The present study investigates whether different verbal risk descriptions accompanying the same statistical data would lead to different decisions and evaluations. Verbal risk descriptions suggested that the medical procedure is more risky (negative valence: “risk is high”/”risk is not low”) or less risky (positive valence: “risk is low”/”risk is not high”). We expect that information with a positive valence (“risk is low”/”risk is not high”) will yield more positive responses than information with a negative valence (“risk is high”/“risk is not low”), but this will be moderated by the language used in the message (indirect vs. direct). Our hypothesis is that direct expressions used to inform about the risk that accompanies the performance of a particular medical procedure with a positive valence (“risk is low”) will facilitate the patient in making a positive decision and will improve the doctor’s evaluation and the patient’s mood evaluation compared to when indirect language is used (“risk is not high”). In contrast, when a message on risk has a negative valence, these effects are expected to be reversed and an indirect expression (“risk is not low”) will facilitate the patient in making a positive decision and evaluations, whereas a direct expression (“risk is high”) will more frequently lead to the rejection of the proposed option and worse evaluations.

Detailed hypotheses are as follows:

### **H1–H3**

When statistical data has positive valence framing (“risk is low”/”risk is not high”), then, according to respondents, patient more frequently decides to undergo a given medical procedure (H1), is in a better mood (H2), and the evaluation of doctor is more positive (H3) as compared to statistical data with negative valence framing (“risk is not high”/”risk is not low”).

### **H4–H6**

When statistical data has negative valence framing and is expressed indirectly (“risk is not low”), then, according to respondents, patient more frequently decides to undergo a given medical procedure (H4), is in a better mood (H5), and the evaluation of doctor is more positive (H6) as compared to direct framing (“risk is high”).

### **H7–H9**

When statistical data has positive valence framing and is expressed indirectly (“it is not high”), then, according to respondents, patient rarely decides to undergo a given medical procedure (H7), is in a worse mood (H8), and the evaluation of doctor is more negative (H9) as compared to direct framing (“it is low”).

## Study 1

### Participants

375 students from the University School of Physical Education participated in this study M_age_ = 21; SD = 1.69; 208 women (55.5%) and 167 men (45.5%); 15% (n = 58) lived in the place with a population up to 1000, 29% (n = 109) up to 50,000; 55% (n = 208) above 50,000. There were 72–81 participants in each experimental condition.

### Procedure

We used a 2 (valence: positive/negative) × 2 (linguistic polarity: direct/indirect) design (Table 1). Participants were informed that the aim of the study was to examine patients’ decisions and evaluations of medical procedures in the case of the risk of side effects. Each participant received two vignettes with two different fragments of a conversation with a patient. The conversations were about the risk of side effects related to performing medical procedures: cordocentesis (situation 1) and echocardiography (situation 2). The presented statistical data regarding the risk of side effects was identical: 10 out of 1000 (situation 1) and 2 out of 1000 (situation 2). In the presented text, before obtaining statistical information about the risk, four types of the verbal description were used (risk is high/risk is not low/risk is low/risk is not high). Participants were randomly assigned to one out of four experimental conditions for each vignette.

**Table 1:** Conditions of the experiment

Conditions		Valence	
		Positive	Negative
Linguistic polarity	Direct	Risk is low	Risk is high
	Indirect	Risk is not high	Risk is not low

The first vignette concerned cordocentesis. According to their condition, participants were given the following text:

Cordocentesis is a diagnostic test that detects the risk of genetic defects by drawing blood from the umbilical cord of the fetus and obtaining fetal blood for further testing. You should be aware that this procedure carries the risk of miscarriage. The risk is high/not high/low/not low. In 10 out of 1000 patients there is a need for immediate termination of pregnancy as a direct result of this medical procedure.

The second vignette had the following text:

Regular physical training leads to changes in the circulatory system. Echocardiography is a very useful procedure in heart assessment. It uses ultrasounds to visualize cardiac structures from transesophageal and transgastric access. Possible complications of echocardiography are primarily related to the process of passing the probe into the esophagus and the upper stomach with the use of local anesthesia and intravenous sedative. The risk of complications is not high/is not low/is high/is low. 2 out of 1000 patients have complications as a direct result of echocardiography.

The messages included real data about the risk of terminating the pregnancy as a result of undergoing cordocentesis (Berry et al. 2013) and the risk related to performing an echocardiography (Daniel et al. 1991). Following this message, participants were asked: *Do you think that the patient will undergo cordoncentesis/echocardiography?* The answers were given on a 5-point scale (1—definitely no, 2—rather no, 3—difficult to say, 4—rather yes, 5—definitely yes). Then, the participants were asked to (1) evaluate the mood of a patient leaving the doctor’s office on a 7-point scale (from 1—in a very bad mood to 7—in a very good mood); and (2) to evaluate the doctor. For evaluation of the doctor, participants indicated whether they would like the doctor who had given this information to be their doctor in the future (from 1—I would definitely not like it, to 7—I would definitely like it) and how reasonably the doctor instructed the patient (from 1—the doctor gave unreasonable information *to the patient to* 7—*the doctor gave reasonable information to the patient*). The alpha coefficient for the two questions about the doctor’s evaluation regarding cordocentesis was: *α *= .77, and regarding echocardiography: *α *= .80. Finally, background information about gender, age and the place of residence was collected.

Participants were informed that the results of the questionnaire were anonymous and would be used for research purposes only. Informed consent was obtained. The study was performed in accordance with the Declaration of Helsinki and approved by the Biomedical Ethics Committee.

### Statistical Analysis

For all experiments, we conducted 2 (valence: positive vs. negative) × 2 (linguistic polarity: direct vs. indirect) multivariate analyses of variance (MANOVA) with patient’s decision, evaluation of mood and evaluation of the doctor as dependent variables. Tables 2 and 3 show the means and standard deviations of these variables per condition for experiments. All analyses were performed using SPSS software v.24.

**Table 2:** Experiment 1a and 1b: mean scores of the decisions and evaluations in the negative and positive valence and language use (direct vs. indirect) conditions

	Positive valence		Negative valence		Neutral^a^
	Direct risk is low M (SD)	Indirect risk is not high M (SD)	Direct risk is high M (SD)	Indirect risk is not low M (SD)	Numbers only M (SD)
	N = 72	N = 80	N = 72	N = 72	N = 76
Cordocentesis					
Decision	2.69 (.88)	2.72 (.93)	2.10 (.82)	2.11 (.81)	2.37 (1.03)^b^
Evaluation of patient mood	3.19 (1.19)	3.00 (1.09)	2.64 (1.01)	2.76 (1.03)	2.63 (1.37)^c^
Evaluation of doctor	9.31 (3.08)	9.67 (2.54)	8.40 (2.94)	8.90 (2.67)	9.08 (2.91)

	N = 72	N = 78	N = 72	N = 81	N = 72
Echocardiography					
Decision	3.72 (.70)	3.83 (.75)	3.04 (.99)	3.18 (1.06)	3.43 (.84)^b^
Evaluation of patient mood	4.78 (1.06)	4.72 (1.18)	3.78 (1.24)	3.90 (1.27)	4.35 (.97)^b^
Evaluation of doctor	10.49 (2.36)	10.63 (2.49)	9.29 (2.76)	9.01 (2.73)	9.83 (2.57)^c^

Higher numbers indicate higher intention to perform a medical procedure, higher expected mood and more positive evaluation of a doctor

^a^In the last column we presented average results for a message without a verbal description. Significant differences between the mean for neutral valence (only numbers) and means for positive valence and negative valence were marked: b—mean for neutral valence significantly higher from the mean of negative value and lower from mean of positive valence with a certainty of *p* < .05; c—mean for neutral valence significantly lower from mean of positive valence and does not differ from mean of negative valence with a certainty of *p* < .05

**Table 3:** Experiment 2: mean values (and standard deviations) of the decision and evaluations in the negative and positive valence and language use (direct vs. indirect) conditions

	Positive valence		Negative valence	
	Direct risk is low M(SD)	Indirect risk is not high M(SD))	Direct risk is high M(SD)	Indirect risk is not low M(SD)
	N = 56	N = 51	N = 67	N = 50
Decision	3.30 (1.01)	3.53 (.97)	2.43 (1.03)	2.48 (1.01)
Evaluation of patient mood	3.16 (1.47)	3.33 (1.16)	2.34 (1.34)	2.58 (1.36)
Evaluation of doctor	8.37 (3.0)	8.65 (3.13)	6.01 (3.31)	7.10 (3.54)

### Results

Both in the first (cordocentesis: 1a) and second situation (echocardiography: 1b) the MANOVA test showed the main effect of the valence (1a: Wilks’ *γ *= .89; *F*(1, 292) = 12.30*; p *< .001*; η*^*2*^= .11; 1b: Wilks’ *γ *= .85; *F*(1, 299) = 17.73; *p *< .001; *η*^*2*^= .15). In both conditions (1a and 1b) subsequent univariate analyses revealed that the valence had a significant effect on the decision [1a: (*F*(1, 292) = 36.16; *p *< .001; *η*^*2*^= .11; 1b: (*F*(1, 299) = 41.83; *p *< .001; *η*^*2*^= .12]; the doctor’s evaluation [1a*: F*(1, 292) = 6.56; *p *< .05*; η*^*2*^= .02; 1b: *F*(1, 299) = 21.22; *p *< .001; η^2^ = .07] and the patient’s mood [1a: *F*(1, 292) = 9.85; *p *< .05; *η*^*2*^= .03; 1b: *F*(1, 299) = 43.76; *p *< .001; *η*^*2*^ = .13]. Participants receiving a verbal description with a positive valance (risk is low/risk is not high) were more positive about a patient’s decision (1a: *M *= 2.71; *SD *= .90; 1b: *M *= 3.78; *SD *= .72), more positively evaluated the doctor (1a*: M *= 9.5; *SD *= 2.81; 1b: *M *= 10.56; *SD *= 2.42) and the patient’s mood (1a: *M *= 3.09; *SD *= 1.41; 1b: *M *= 4.75; *SD *= 1.12) than the participants receiving a verbal description with a negative valence (decision 1a: *M *= 2.10; *SD *= .82; 1b: *M *= 3.12; *SD *= 1.03; evaluation of the doctor: 1a: *M *= 8.65; *SD *= 2.81; 1b: *M *= 9.14; *SD *= 2.85; mood 1a: *M *= 2.7; *SD *= 1.02; 1b: *M *= 3.84; *SD *= 1.25). We did not observe neither the main effect of linguistic polarity in any situation [1a: Wilks’ *γ *= .99; *F*((1, 292) < 1; 1b: Wilks’ *γ *= .99; *F*(1, 299) < 1] nor any interaction between the valence and linguistic polarity on psychosocial outcomes [1a: Wilks’ *γ* = .99, *F*(1, 292) < 1; 1b: Wilks’ *γ* = .99, *F*(1, 299)) < 1]. Therefore, we confirmed hypotheses H1–H3, not H4–H9.

## Study 2

Due to the fact that some researchers point out that studies on young people cannot be generalized onto other age groups, particularly in the area of health (Gong et al. 2013), the second study was carried out only on adults.

### Participants

The study was carried out among 224 adults aged 40-90 years (*M*_*age*_= 57; *SD *= 11.82); 134 women and 90 men. The level of education: 19% (N = 43) had vocational education; 38% (N = 85) high-school education; 43% (N = 96) undergraduate and graduate education. Habitation: 13% (N = 30) lived in a place up to 1000 citizens; 29% (N = 65) up to 50,000; 58% (N = 129) above 50,000. The four frames were similar with respect to the distribution of gender (*χ*^2^(3, 224) = 5.73, *ns*), mean age (*F*(3, 220) < .53, *ns*), education (*χ*^2^(6, 224) = 4.97, ns). The eligibility criteria were as follows: (a) age above 39 years, (b) no history of serious mental disorder, (c) no medical condition that would affect the ability to participate. There were 50–67 participants in each experimental condition.

### Procedure

We used an identical design: 2 (valence: positive/negative) × 2 (linguistic polarity: direct/indirect). All participants were volunteers. Participants were presented with a written excerpt of a conversation in which a patient was informed about the risk associated with one of the most serious complications following diagnostic colonoscopy: perforation. The message included real statistical data on the risk of perforation associated with conducting a colonoscopy (1:1000) (Lüning et al. 2007). Like in the first study, we manipulated the valence (positive/negative) and the linguistic polarity (direct/indirect). In the presented text, before obtaining statistical information about the risk, four types of verbal descriptions were used (risk is high/risk is not low/risk is low/risk is not high). After reading a hypothetical situation, the participants were asked the same questions as in the first study. Reliability for the measures of doctor’s evaluation was again satisfactory (*α *= .90).

### Results

As it was the case in the study conducted among students, no main nor interaction effects with linguistic polarity were found. The MANOVA test showed only the main effect of the valence [Wilks’ *γ *= .81; *F*(1, 220) = 16.59; *p *< .001; *η*^*2*^= .19]. Valence had a significant effect on the decision [*F*(1, 220) = 50.14; *p *< .001; *η*^*2*^= .19]; doctor’s evaluation [*F*(1, 220) = 19.99; *p *< .001; *η*^*2*^= .08], and the patient’s mood evaluation [*F*(1, 220) = 18.93; *p *< .001; *η*^*2*^= .08]. Similarly to the first study, participants receiving verbal descriptions with a positive valence (“risk is low”/”risk is not high”) were more positive about a patient’s decision (*M *= 3.41; *SD *= .10), evaluated more positively: the doctor (*M *= 8.50; SD = 3.05) and the patient’s mood (*M *= 3.24; *SD *= 1.33) than participants receiving verbal descriptions with a negative valence (“risk is high”/”risk is not low”): decision: *M *= 2.45; *SD *= 1.02; evaluation of the doctor: *M *= 6.48; *SD *= 3.44; mood: *M *= 2.44; *SD *= 1.35).

As in the first study, we confirmed hypotheses H1–H3, but we did not confirm hypotheses H4–H9.

## General Discussion

The aim of this study was to indicate how verbal descriptions of the risk of side effects with different valence (positive vs. negative) and language (direct vs. indirect) accompanying identical statistical data can influence patients’ evaluation and making medical decisions. We received interesting results. First of all, the results replicate previous research, indicating that verbal descriptions accompanying numeric data have a significant impact on medical decisions made by the patients (Knapp et al. 2015; Büchter et al. 2014). Results of this study prove that verbal descriptions accompanying statistical data could not only change the patient’s willingness to undergo medical procedures but also influence the evaluation of a doctor and the patient’s mood. Previous studies have shown that people tend to focus on the qualitative aspects of risks instead of, and in addition to, the quantitative aspects (Visschers et al. 2009). This may be due to the fact that people need help in interpreting not only what the numbers are but what they actually mean. Although there are some known biases towards attaching verbal information to numbers (Trevena et al. 2013), presenting information in this way seems easier, more neutral and personal (Wallsten et al. 1993) and, as our results indicate, people frequently rely on it.

Subsequently, as was found by others, the difference in the valence of the description has influenced patients’ evaluation and making medical decisions (Welkenhuysen et al. 2001). Our experiments have shown that the same probability statements can lead to different evaluations depending upon the way they are phrased (as rather positive or negative). This finding could be attributed to the affective associations evoked by each frame. By expressing probability verbally, we are suggesting and supporting either a positive or a negative conclusion (Teigen and Brun 1999). Levin et al. (1998) suggested that presenting an object or an event in a positive or negative framing affects information processing in a way similar to priming. The activation of favorable or unfavorable associations biases people’s judgments and evaluations because information is encoded relative to its descriptive valence, causing valence-consistent evaluation shifts (Levin et al. 1998; Sher and McKenzie 2006). It seems that verbal descriptions accompanying statistical data defining the risk as not significant (low or not high) evoked less negative affect than those describing the risk as significant (high or not low), which subsequently led to positive attitude toward undergoing a particular medical procedure.

The hypotheses on the mitigating role of indirect expressions were not confirmed. Direct negative terms are indistinguishable from indirect negative terms, and direct positive terms have the same meaning as indirect positive terms. Our results are consistent with the results obtained by Colston (1999), who proved that the asymmetry of meaning appeared only towards events in which people expected positive results. However, when the event was described in a way that led people to expect a negative outcome (negative expectations), there was no asymmetry and the differences between direct and indirect terms did not appear (“difficult” = “not easy”, “easy” = “not difficult”). This study concerned the risk associated with the incidence of side effects of medical procedures, so it is similar to experiments by Colston, regarding negative expectations. The message not only concerned potential side effects but also the word “risk” as having negative connotations (Rozin et al. 2010). The obtained results are generally in line with the literalist view of semantics (Colston 1999), where negation simply provides an alternative way of saying the same thing as direct term (e.g., “not wet” = “dry”). Due to the fact that this study did not include situations in which the interlocutor would expect a positive message, the obtained results do not allow to unambiguously settle in favor of any theory on the relations between direct and indirect terms of different valences discussed in the introduction. They only confirm Colston’s results that when the interlocutor expects negative information, the mitigating role of indirect formulation may not occur.

An alternative explanation for the lack of differences between direct and indirect messages may result from the fact that the framing effect in the area of health is of little importance (Akl et al. 2011). What can moderate the framing effect is the patient’s involvement or his/her strong a priori approach (Levin et al. 1998; Kerler et al. 2012). Studies show that if the scenario is highly related to the participants, the framing effect frequently diminishes or even disappears (McElroy and Seta 2003). While medical situations are filled with emotional involvement, the effect of framing is minimal. Therefore, no differences between direct and indirect wording may result from the fact that medical profession is filled with emotional load.

Some limitation should be examined carefully. The study presented a hypothetical scenario, which is its potential weakness. Despite the fact that the study was designed to provide real data utilized in real world decisions and that we used situations that may be encountered by young people (study 1) or adults (study 2), all the presented cases were fictitious. Moreover, for ethical reasons, respondents were asked to assess the situation from the perspective of a patient in a given situation, rather than to imagine themselves in that situation even though the studies clearly indicate that empathy is stronger when in self-perspective than in others-perspective (Jackson et al. 2006). It may have resulted in lower respondents’ involvement in the study, and thus influence the results obtained.

It should be noted that the obtained results might be related to the probability of occurrence of adverse effects in the examined situations. Due to the fact that in these situations the probability of side effects was low (1 to 10 in 1000 people), the conclusions based on the research should be limited to situations in which the risk is low. In the future, it should be verified whether the results obtained do not refer only to situations in which the risk is at such a low level. It is possible that data expressing a clearly high risk of side effects (e.g., 5 out of 10 people) would be more resistant to context effects, i.e. in this case the verbal label accompanying the figures. This, however, requires verification in subsequent studies that should cover not only high and low risk situations, but also different types of risks (e.g., life-threatening, no life-threatening but permanent consequences, more tolerable and temporary consequences, etc.).

Due to the low sample size the generalizability of our results is also limited. Additionally, the study was conducted in Polish, therefore, the effects may be limited to the tested language. Another limitation of this research was the lack of demographic diversity in our samples. The participants were a convenience sample. Despite the fact that in the study 2 the participants were not students but adults, this sample was more highly educated than the general population, which could have contributed to the higher levels of performance in the study. In the future, such studies should use representative samples or be conducted in real life settings and measure potentially more relevant outcomes, i.e. only people’s behavior. On a more critical note, it is questionable whether the obtained results may be compared to Colston’s study, since the author asked participants what they thought a speaker meant to express, given a particular scenario, and thus, to reflect on the intended meaning of the speaker. Therefore, participants of this study and the study by Colston faced completely different tasks.

The current study does not support the fact that indirect wording weakens messages about risk and highlights the strength and persistence of following verbal descriptions, particularly their affective valences. The valence related to the size of risk impacts judgment regardless of whether or not the message is presented directly or indirectly. Doctors should realize that they influence patients’ decisions by selecting specific word labels to present possible side effects related to performing medical procedures. What is particularly important in the decision-making process is the affective meaning of verbal descriptions. Patients frequently expect that they will receive verbal descriptions of possible side effects, but because finding emotionally neutral words is challenging we should carefully consider whether or not to use them.
